# Speckle statistics as a tool to distinguish collective behaviors of Zebrafish shoals

**DOI:** 10.1038/s41598-024-64229-8

**Published:** 2024-07-09

**Authors:** Adauto J. F. de Souza, Antonio. R. de C. Romaguera, João V. A. Vasconcelos, Luis G. Negreiros-Neto, Viviane M. de Oliveira, Pabyton G. Cadena, Anderson L. R. Barbosa, Marcelo L. Lyra

**Affiliations:** 1 Departamento de Física, Universidade Federal Rural de Pernambuco, Recife, PE 52171-900 Brazil; 2 Instituto de Física, Universidade Federal de Alagoas, Maceió, AL 57072-970 Brazil; 3 Departamento de Morfologia e Fisiologia Animal, Universidade Federal Rural de Pernambuco, Recife, PE 52171-900 Brazil

**Keywords:** Statistical methods, Population dynamics

## Abstract

Zebrafish have become an important model animal for studying the emergence of collective behavior in nature. Here, we show how to properly analyze the polarization statistics to distinguish shoal regimes. In analogy with the statistical properties of optical speckles, we show that exponential and Rayleigh distributions emerge in shoals with many fish with uncorrelated velocity directions. In the opposite limit of just two fish, the polarization distribution peaks at high polarity, with the average value being a decreasing function of the shoal’s size, even in the absence of correlations. We also perform a set of experiments unveiling two shoaling regimes. Large shoals behave as small domains with strong intra-domain and weak inter-domain correlations. A strongly correlated regime develops for small shoals. The reported polarization statistical features shall guide future automated neuroscience, pharmacological, toxicological, and embryogenesis-motivated experiments aiming to explore the collective behavior of fish shoals.

## Introduction

Zebrafish (Danio rerio) is a vertebrate model animal of increasing relevance over the last few years. The possibility of laboratory breeding in large numbers with controlled genetics, as well as its clear embryo allowing optical imaging at the organism level^1,2^, gives some advantages of zebrafish over rodent models for embryogenesis studies^3–5^. Moreover, 84% of the genes known to be associated with human diseases have a homolog in zebrafish, an aspect that has prompted several scientific studies aimed at comprehending the basic mechanisms driving developmental and brain disorders, as well as metabolic diseases^6–10^.

Zebrafish, like the majority of fish species, exhibit social behavior with a strong tendency to live in groups, usually termed as shoals^11,12^. This can benefit the group by promoting an improved capability to avoid predators, obtain food, and explore new environments when identifying proper migratory routes. However, there is a higher competition for food in large groups. Further, large groups are more easily identified by predators. Therefore, these competitive benefit and cost aspects of shoal formation have to be balanced and ultimately determine the optimal shoal size. There are two main classes of fish groups. They can aggregate, thus developing strong correlations in their positions without significant correlations in their velocity orientations. This kind of group is usually simply termed a shoal. In fish schools, relevant correlations are also developed in their velocity directions, promoting a synchronized motion of the group. To differentiate shoal and school collective behavior, the tendency of individuals to adopt the same orientation is usually quantified through group polarization defined as the magnitude of the average unit direction of all fish^13–22^.

The social behavior of zebrafish has been recently explored in several branches of biosciences. For example, learning and memory behavioral tests have been recently performed using visual and sound stimulous^22–24^, evidencing a lower polarization degree of stimulated fish. Toxicological studies using drugs of abuse have also pointed out that nicotine has a strong impact on shoal cohesion while alcohol disrupts significantly the group polarization^20^. The tendency of shoaling has been shown to increase with age^21^ while polarization decreases when fish become habituated to the environment in experiments performed in test tanks^19^. Zebrafish shoals have also been used to study brain disorders impacting social behavior such as stress, Alzheimer’s disease, and the autism spectrum disorder^25–28^. Variations in group cohesion and polarization signal changes in the social ability related to specific neurological diseases, pharmacological interventions, and toxicological and environmental influences.

Fish polarization is the standard quantity used to distinguish the simple shoaling behavior from the most correlated schooling formation. However, the polarization time series depicts strong fluctuations. The polarization distribution function (PDF), the average value, and the standard deviation are, therefore, usually considered the main quantifiers to infer the degree of orientationally synchronized motion. Peaks in the PDF at small and large polarizations are associated with signatures of shoaling and schooling, respectively. Also, a time interval on which the fish present a large polarization is assumed to be a schooling period, while the intervals with the opposite small polarization are interpreted as shoaling periods^19^. Further, some studies have reported a substantial decrease in the average polarization with the number of fish, with the PDF peak moving towards smaller polarization values^19,22^. This feature has been directly associated with a decreasing capability of schooling.

Some of the above statements relating polarization features with the tendency to develop schooling behavior lack the support of a solid statistical framework. Some fundamental questions deserve a deeper understanding. What is the expected polarization PDF for the case of pure shoals? How do the polarization PDF, average, and standard deviation depend on the shoal’s size? Do peaks in the PDF can be directly related to shoaling and schooling trends? In this work, we address the above questions. Considering that the fish polarization in pure shoals with no orientational correlations results from the sum of random phasors with the same length, we draw a direct analogy with the speckle distribution of the optical field and provide the exact polarization PDF for shoals with *N* fish.

Speckle is the term given in optics to the granular intensity pattern observed at a spot when coherent laser light is reflected by a surface having some degree of roughness on the scale of the light’s wavelength^28^. The granularity of the light intensity depicts typical fluctuations in space even when the spot illumination is relatively uniform. A similar speckle pattern is observed when laser light is transmitted through media with diffusers such as particle suspensions. It affects the ability to extract information contained in an image. This phenomenon is generally observed when wave fields are transmitted by or reflected from rough objects, finding application in radar microwave imaging and ultrasound medical imaging.

Exploring the analogy between the fish group polarization and optical speckles, we compare the pure shoal prediction with recently published data from an experiment with zebrafish confined to a test tank. Further, we will present data from a new experimental setup showing that groups with many fish behave as a shoal with short-range polarization correlations. In contrast, small groups exhibit a distinct finite-size dependence, unveiling the schooling scaling behavior.

## Results

**Figure 1:**
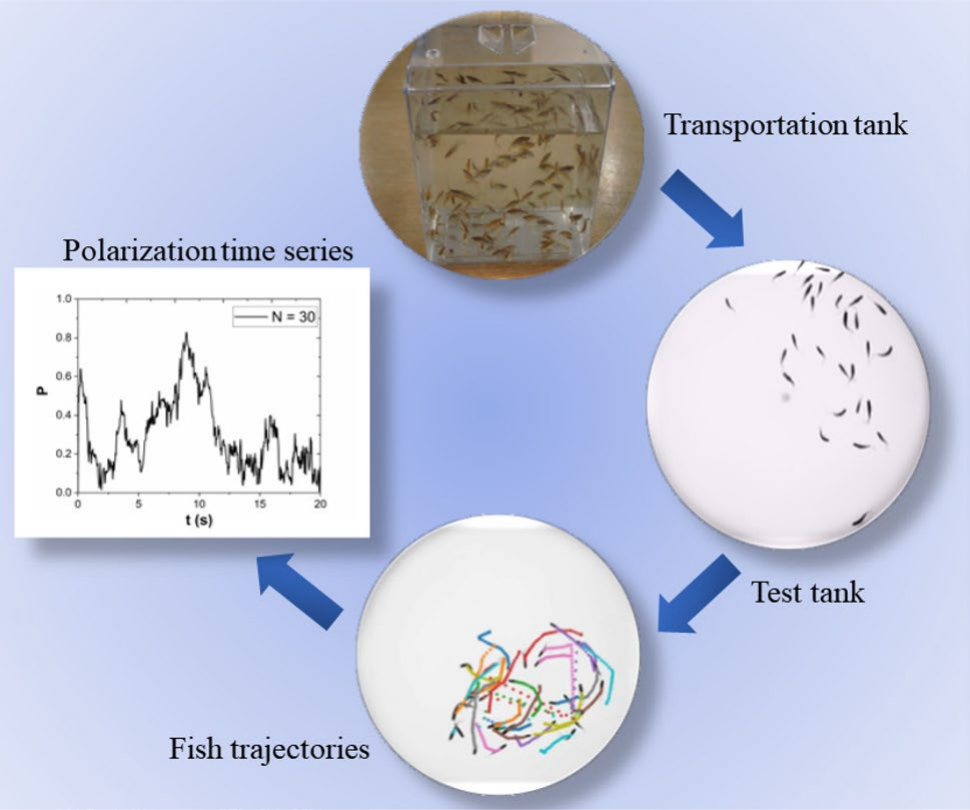
Schematic representation of the experimental protocol: Fish are brought from the vivarium to the laboratory in a transportation tank. A group is selected at random and placed in the test tank. Images of the fish shoal are captured by a camera and digitalized using the IdTracker software. Fish trajectories are recorded and the shoal’s polarization time series are extracted.

In the present work, we focused on the statistical properties of the polarization to explore the possible tendency of the fish to align. The sequence of processes and the experimental protocol are detailed in Fig. 1. The average velocity director in a shoal with *N* fish is defined as

1 $$\begin{aligned} \textbf{u}(t) = \frac{1}{N}\sum _{i=1}^n \textbf{u}_i(t) = u^x(t)\hat{e}_x+u^y(t)\hat{e}_y , \end{aligned}$$

where $\textbf{u}_{i}(t) = \textbf{v}_i(t)/|\textbf{v}_i(t)| = u^x_i(t)\hat{e}_x + u^y_i(t)\hat{e}_y$ is the direction of the velocity vector $\textbf{v}_i(t)$ of the *i*-th fish in the shoal. $\hat{e}_x$ and $\hat{e}_y$ correspond to the unitary director vectors in a Cartesian coordinate fixed reference frame. The shoal’s polarization at time *P* is defined as the magnitude of the average director as $P(t)=|\textbf{u}_t|$. The polarization fluctuates in time as the fish shoal moves. Its probability distribution function and the associated statistical quantifiers, such as the average value and standard deviation, can provide important signatures of possible underlying correlations within the fish shoal.

### Fully uncorrelated shoals

Before analyzing the polarization statistics of the recorded experimental data aiming to identify possible correlations in the fish velocity directions, it is essential to have in mind the expected probability distributions and corresponding quantifiers for the limiting case of fully uncorrelated shoals. The complete statistical framework is summarized in Table 1 (details in section “Methods”). The polarization statistics is analogous to the optical speckle statistics. Numerical evaluation of the PDFs depicts a very slow convergence due to the highly oscillatory nature of the integrand. Alternatively, a Monte Carlo numerical procedure can provide very precise estimates for these PDFs and the corresponding statistical quantifiers.

**Table 1:** Statistics of *P* and $P^2$ for shoals with uncorrelated velocity directions: statistical characteristics of the polarization *P* and its square $P^2$ for shoals with *N* fish with fully uncorrelated velocity directions.

	*P*	$P^{2}$
Mean	$2/\pi$ ($N=2$)	
		1/*N*
	$\sqrt{\pi /4}/N^{1/2}$ ($N\gg 1$)	
Standard	$\sqrt{(\pi ^2-8)/(2\pi ^2)}$ ($N=2$)	
deviation		$\sqrt{1-1/N}/N$
(SD)	$\sqrt{(4-\pi )/4}/N^{1/2}$ ($N\gg 1$)	
Mean/SD	$\sqrt{8/(\pi ^2-8)}$ (N=2)	
		$$\sqrt{1-1/N}$$
	$\sqrt{\pi /(4-\pi )}$ ($N\gg 1$)	
PDF	$P\int _0^{\infty }u J_0^N(u)J_0(Pu)du$	$\frac{1}{2}\int _0^{\infty }u J_0^N(u)J_0(Pu)du~$
	$\frac{2}{\pi \sqrt{1-P^2}}$ ($N=2$)	$\frac{1}{\pi \sqrt{P^2(1-P^2)}}$ ($N=2$)
	$2NPe^{-N P^2}$ ($N\gg 1$)	$N e^{-NP^2}$ ($N\gg 1$)

Mean, standard deviation (SD), the corresponding ratio, and the probability distribution functions (PDF) for both polarization measures are shown. Rayleigh (*P*) and exponential ($P^2$) PDFs for $N\gg 1$ follow from the central limit theorem. $J_0(u)$ is a Bessel function of the first kind.

**Figure 2:**
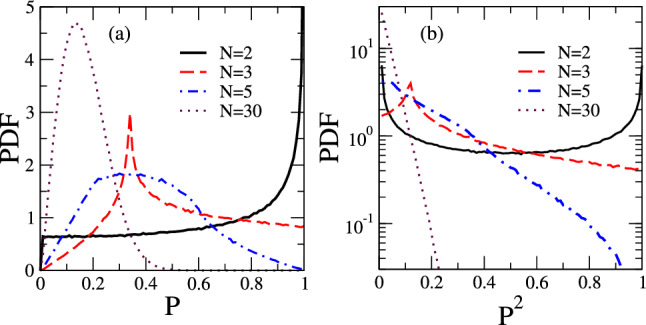
PDFs of *P* and $P^2$ for uncorrelated velocity directions: probability distributions functions (PDF) for the polarization *P* (**a**) and its square $P^2$ (**b**) obtained from Monte Carlo calculations for uncorrelated random velocity directions of the fish. For $N=2$ and $N\gg 1$, the PDFs coincide with the analytical calculations, evolving for Rayleigh (*P*) and exponential ($P^2$) distributions as *N* increases (see text). Peaks appearing for small shoals are casual and not related to any tendency of fish alignment.

**Figure 3:**
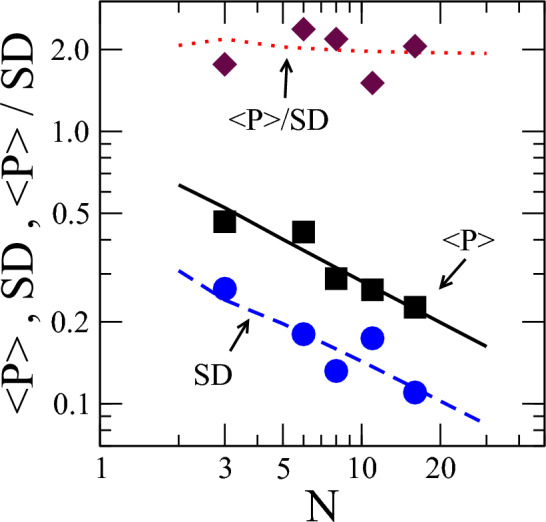
Size dependence of statistical quantifiers for shoals with uncorrelated velocity directions: statistical quantifiers $\langle P\rangle$ (solid line), its standard deviation SD (dashed line), and the ratio $\langle P\rangle / SD$ (dotted line) from Monte Carlo calculations considering fish shoals with uncorrelated velocity directions. Asymptotic values for $N=2$ and $N\gg 1$ are in full agreement with the analytical results. Experimental data from ref.^22^ for $\langle P\rangle$ (squares), its standard deviation SD (circles), and the ratio $\langle P\rangle / SD$ (diamonds) are also included. These data indicate that the experimental setup used in Ref.^22^ produced shoals with no significant correlation in the fish velocity directions.

**Figure 4:**
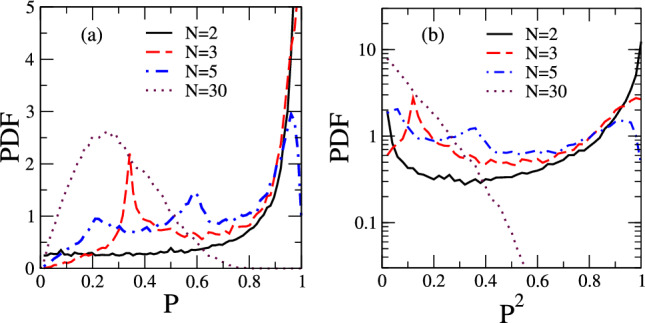
Experimental PDFs for *P* and $P^2$: probability distribution functions for *P* (**a**) and $P^2$ (**b**) raised from the experimental data of the shoal’s polarization time series for the same number of fish used in Fig. 2 for fully uncorrelated shoals. For small *N* there is a clear tendency towards larger polarizations. For large *N* the PDFs evolve towards Rayleigh (PDF for *P*) and exponential (PDF for $P^2$) but with widths larger than in the corresponding PDFs for uncorrelated shoals with the same number of fish.

**Figure 5:**
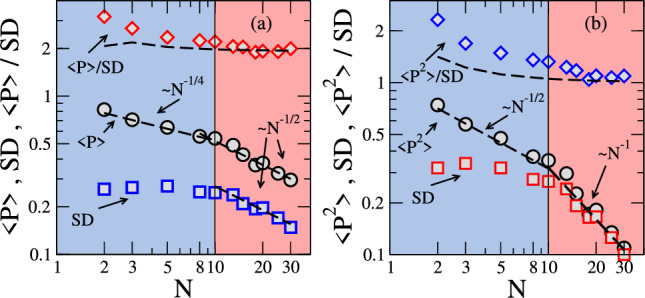
Statistical quantifiers for *P* and $P^2$ from experimental data: statistical quantifiers (average over time and the five trails, the standard deviation SD, and their ratio) for *P* (**a**) and $P ^2$ (**b**) extracted from the experimental data. SD measures the variability of the polarization time series, acting as a noise estimator. Error bars concerning the variability of the average polarization measures among trials are of the order of the symbols’ size. Two shoal regimes can be identified. For $N >N_c \simeq 10$ the finite-size scaling laws have the same exponents predicted for uncorrelated shoals but with larger coefficients. A new shoaling regime emerges for $N <N_c \simeq 10$ with slower power-laws for the averages and roughly size-independent SDs.

The resulting PDFs for some representative values of *N* are shown in Fig. 2. Notice that for $N=2$ both PDFs for *P* and $P^2$ depict a diverging peak at $P=P^2=1$ even in the full absence of correlations. Therefore, the presence of a peak in the polarization distribution at $P=1$ can not be uniquely associated with a tendency of the fish to swim in the same direction but rather to a casual alignment. Further, $P^2$ has a PDF with a peak at $P^2=0$ (absent in the PDF of *P* at $P=0$). Such a peak does not indicate a tendency of the fish to swim in opposite directions, being also casual. These peaks are a direct consequence of the fact that when the relative angle $\theta _{1,2}$ between the fish is randomly and uniformly oriented, $\cos {\theta _{i,j}}$ is not uniformly distributed. The peak at $P=P^2=1$ is suppressed for $N>2$. As *N* increases, the distributions evolve towards the Rayleigh distribution for *P* and towards the exponential distribution for $P^2$. The above distributions are the same ones found for the electric field magnitude and intensity of optical speckles^28^.

In Fig. 3, we plot the statistical quantifiers $\langle P\rangle$, its *SD*, and $\langle P\rangle / SD$ obtained from the Monte Carlo calculations (solid, dashed, and dotted lines, respectively). The results fully agree with the analytical calculations. In Fig. 3, we also include data for the average polarization and its SD from a recent experiment with zebrafish shoals designed to study behavioral changes due to external stimuli^22^. In this experiment, fish were placed in an opaque glass tank with dimensions $550\times 200\times 250$ mm with oxygenation and feeding compartments. The polarization data of freely swimming fish (with no external stimuli) are very close to the above prediction for shoals with no correlations in the velocity directions. One can conclude that the experimental setup used in Ref.^22^ favors the formation of shoals where, although the fish tend to aggregate, they do not develop any significant correlation in their swimming directions.

### Polarization statistics: experimental data analysis

We extracted the time series of the shoal’s polarization from the digitalized data of the individual fish trajectories (see a video S1 in the supplementary material). The PDFs of the polarization and its square were raised using data from five trials for each shoal size studied. These are reported in Fig. 4.

The PDFs from the experimental data present similarities with those for shoals with fully uncorrelated velocity directions, as previously discussed. However, some trends signal the presence of relevant correlations. Focusing initially on the $N=2$ experiment, the PDF for *P* peaks at $P=1$ and develops a plateau for small polarization values (see Fig. 4a). Although this overall behavior is also depicted for uncorrelated shoals (see Fig. 2a), the plateau is lower for the experimental data while the peak at $P=1$ is more pronounced. This feature indicates a tendency towards larger polarizations in comparison with uncorrelated shoals. Such bias becomes more evident in the PDF of $P^2$ (Fig. 4b). The peaks at $P^2=0$ and $P^2=1$ are quite asymmetric, favoring high polarizations. This contrasts with the symmetry predicted for uncorrelated shoals (Fig. 2b). The PDFs peak at $P=P^2=1$ for $N=3$, a feature not present in the absence of correlations. For large shoals ($N=30$ is illustrated in Fig. 4) the PDFs for *P* and $P^2$ assume, respectively, the Rayleigh and exponential forms predicted for uncorrelated shoals. However, they are much wider than the corresponding uncorrelated PDFs with the same number of fish, unveiling that fish orientations are not completely uncorrelated in the experiments performed, even in the regime of large shoals.

The size dependence of the main statistical quantifiers (average, standard deviation, and their ratio) of *P* and $P^2$ were also extracted from the experimental data to allow for a more direct comparison with the analytical and Monte Carlo results derived for uncorrelated shoals. Results are shown in Fig. 5 for *P* and $P^2$. The data signal for two distinct shoaling regimes:

(a) For shoals with $N> N_c\simeq 10$ fish, the finite-size scaling exponents are the same as predicted for uncorrelated shoals, namely $\langle P\rangle \propto 1/N^{1/2}$ (as well as its SD) and $\langle P^2\rangle \propto 1/N$ (and its SD). Their ratios also coincide with the prediction for uncorrelated shoals $\langle P\rangle / SD = 1.913...$ and $\langle P^2\rangle / SD = 1$. However, the coefficients of the finite-size scaling laws are larger. For example, $\langle P^2\rangle = n/N$ with $n\simeq 3.2$, instead of $n=1$ holding for fully uncorrelated shoals. A similar rescaling coefficient of the shoal size is found for the SD of $P^2$. Therefore, the effective number of uncorrelated variables is $N^*=N/n$. A possible interpretation is that the shoal in this regime is composed of domains of average size $n\simeq 3$ with strong orientational correlations within each domain but weak inter-domain correlations. Such a picture is also consistent with the data for $\langle P\rangle$. It is important to stress that this interpretation is also consistent with a recent study of zebrafish shoals^29^ where the authors showed fish to interact predominantly with their nearest neighbors in groups of three fish, perceiving the rest of the group as a fluctuating background.

(b) For shoals with $N< N_c\simeq 10$ fish, new finite-size scaling laws emerge: $\langle P\rangle \propto 1/N^{1/4}$ and $\langle P^2\rangle \propto 1/N^{1/2}$. The corresponding SDs depict a weak size dependence. As a result, the ratios between average and SD strongly deviate from the prediction for uncorrelated orientations. It is instructive to focus on the finite-size dependence of $\langle P^2\rangle$ for which the prediction for fully uncorrelated shoals is exactly 1/*N* for all shoal sizes. The experimental data in the small shoals’ regime is well fitted by $\langle P^2\rangle = 1/N^{1/2}$, unveiling non-trivial orientational correlations. The characteristic shoal size separating the above two regimes is the one for which the above scaling laws, namely $\langle P^2\rangle = n/N$ for $N>N_c$ and $\langle P^2\rangle = 1/N^{1/2}$ for $N<N_c$, provide the same value for $\langle P^2\rangle$. This implies $N_c=n^2$, consistent with the reported crossover in the experimental data. A change in the shoaling regime has also been reported in experiments with young fish (Oreochromis niloticus L.), signaled by changes in the density dependence of the most probable inter-distance and width of the relative orientation distribution^30^.

## Discussion

We reported measurements of the polarization time series of zebrafish shoals confined in a shallow circular tank. Shoal sizes ranging from $N=2$ up to $N=30$ fish were monitored through an automated system. We also provide analytical results for the polarization statistics for the case of pure shoals on which fish velocity directors are completely uncorrelated. The polarization probability distribution function was shown to be analogous to the one exhibited by optical speckles resulting from the superposition of electric field vectors scattered by a rough surface. The polarization PDF can be analytically written in integral form. These can be accurately drawn either from numerical integration algorithms or Monte Carlo calculations. For large shoals, the central limit theorem implies a Rayleigh distribution for the polarization and an exponential distribution for its square. The PDF’s of small uncorrelated shoals present peaks, evidencing that they can not be directly associated with a tendency of shoaling or schooling. The resulting statistics accurately fit data from a recent experiment with zebrafish shoals in a training setup^22^, indicating that it does not produce significant correlations in the velocity directors. This finding is in line with previous experiments showing that the fish habituation to the test tank environment reduces the shoal’s polarization^19^.

In our experiment, polarization data were extracted after a short acclimatization period of fish in the test tank, avoiding the habituation effect. We showed that relevant differences in the polarization statistics emerge when compared to that expected for fully uncorrelated shoals. For shoals with a large number *N* of fish, the PDF’s have the predicted Rayleigh and exponential forms, but with a much larger width. In this regime, the average polarization decays as $1/\sqrt{N}$ and its square as 1/*N*. Similar laws are found for the standard deviations, thus providing a finite limiting value for the ratio between the average and corresponding deviation. However, the distribution widths are consistent with that of uncorrelated shoals with a smaller number of fish *N*/*n* with $n\simeq 3.2$. As such, the shoal behaves as being composed of a set of *N*/*n* uncorrelated clusters, each one being, on average, composed of *n* strongly correlated fish. Small shoals depict a new scaling regime, with the average polarization decaying as $1/N^{1/4}$ and average squared polarization as $1/\sqrt{N}$. The standard deviations become weakly size-dependent. These new laws signal the presence of non-trivial correlations and characterize the schooling regime.

The presently reported change from shoaling to schooling when the number of fish decreases opens many directions for future studies. For example, groups of similar size will likely exhibit different behaviors if confined to a smaller area or allowed to roam a larger one. Does the fish density in confined geometries impact the crossover between the reported regimes? Are confinement-related quantities such as the average interindividual and the wall distances relevant? Other relevant questions are also worth investigating. How do the test tank geometry, habituation time, water temperature, age, and gender ratio affect the typical crossover shoal size? Can this characteristic shoal size be manipulated genetically, by training and memory protocols, or through pharmacological interventions focused on social disorders? Does it change due to the exposure to water contaminants and drugs of abuse? Theoretical modeling of the social forces relevant to the emergence of collective behavior in zebrafish would be valuable to shed light on the underlying mechanisms leading to the observed change in the scaling behavior of the average polarization with the group size. Attractive, repulsive, orientational, and velocity matching interactions between individuals, the interaction of individuals with the walls, as well as propulsive, viscous, and stochastic forces, can be extracted using advanced data-driven methods^35–40^ and would allow the identification of the relevant force-field parameters governing the distinct scaling regimes. The reported theoretical polarization distribution functions of uncorrelated shoals shall guide future experimental and theoretical works aiming to explore the social behavior of fish and to elucidate the biological and physical mechanisms behind the development of complex collective behavior in nature.

## Methods

### Experimental methodology

The experimental procedure we implemented to study the collective swimming regimes of zebrafish shoals was approved by the Ethics Committee of UFRPE for the Use of Animals, protocol number 7373131021, which is in accordance with ARRIVE guidelines. A total of 115 healthy adult zebrafish were used that have been raised and housed in the vivarium condition according to OECD 236 (2013) guidelines^31^. Wild-type adult fish (1 year old) were raised and kept in the vivarium. The zebrafish were housed under controlled laboratory conditions: maintaining a temperature of 26 ± 1 ^∘^C, pH levels at 7.5 ± 0.5, and a light-dark cycle of 14/10 hours. Water was partially replaced once a week, and parameters such as dissolved oxygen, ammonia, nitrite, and nitrates were consistently monitored and maintained within acceptable ranges. The fish were fed a diet consisting of fish feed (with 30% crude protein) two times daily and brine shrimp (Artemia ssp.) once daily^32^. The animals had one and a half years of age and had an average body size of 3.0 cm. The fish were fed at 7 am, 12 pm, and 4 pm, while the experiments occurred in the morning, between 8 am and 12 pm, Brazil time.

The fish were moved from the vivarium to the laboratory in a proper transportation tank. A certain number *N* of them was randomly chosen and gently poured in a circular tank with a diameter of 50cm and water height of 5cm kept at a temperature of 24.5 ^∘^C. The tank was uniformly illuminated from the bottom by a diffuse LED light source with a square shape measuring $60\times 60$ cm. The tank was surrounded by Styrofoam walls, which ensured diffuse lighting conditions to reduce environmental disturbances from the outside. The fish were left in the tank for 10 min to acclimate. Their trajectories were recorded during 8.5 min by a camera positioned 1.46 m above the tank and digitalized using the idTracker software^33^. The first and last minutes were cut from the recordings before implementing the statistical analysis.

We performed experiments with different numbers of fish in the tank (N = 2, 3, 5, 8, 10, 13, 15, 18, 20, 25, 30). Five trials were done for each value of *N*. After each trial, the fish were removed from the tank. Water was replaced before a new group of fish was used in the next trial.

### Statistics of fully uncorrelated shoals

We start by analyzing the statistics of the squared polarization

2 $$\begin{aligned} P^2(t)= & {} |\textbf{u}(t)|^2= \textbf{u}(t)\centerdot \textbf{u}(t) \nonumber \\ {}= & {} \frac{1}{N^2}\left[ \sum _i|\textbf{u}_i(t)|^2+\sum _{i\ne j}\textbf{u}_i(t)\centerdot \textbf{u}_i(t)\right] . \end{aligned}$$

In the above expression $\textbf{u}_i(t)\centerdot \textbf{u}_i(t) = \cos {\theta _{ij}}$ where $\theta _{ij}$ is the angle between the velocity directions of fish *i* and *j*. For fully uncorrected directions, its average value vanishes. On the other hand $|\textbf{u}_i(t)|^2 = 1$. Therefore, the average squared polarization and its standard deviation can be directly obtained as

3 $$\begin{aligned} \langle P^2\rangle= & {} \frac{1}{N} \end{aligned}$$

4 $$\begin{aligned} SD= & {} \sqrt{\langle P^4\rangle - \langle P^2\rangle ^2} = \frac{1}{N}\sqrt{1-\frac{1}{N}}, \end{aligned}$$

with the ratio $\langle P^2\rangle / SD = 1/\sqrt{1-1/N}$ being $\sqrt{2}$ for $N=2$ and 1 in the regime of $N\gg 1$.

The own probability distribution function (PDF) of $P^2$ has simple expressions in limiting cases. For a shoal composed of just $N=2$ fish, the squared polarization is just $P^2 = (1+\cos {\theta _{1,2}})/2$. Considering $\theta _{1,2}$ being uniformly distributed for uncorrelated swimming directions, one has

5 $$\begin{aligned} \text {PDF}(P^2) = \frac{1}{\pi \sqrt{P^2(1-P^2)}} ; ~~~(N=2) . \end{aligned}$$

In the opposite regime of shoals with $N\gg 1$ fish, the components of $\textbf{u}(t)$ are sums of independent random variables associated with the Cartesian components of each velocity direction. The Cartesian components of each fish velocity direction have null average values and standard deviation $\sqrt{\langle \cos {\theta _i}^2\rangle }=\sqrt{\langle \sin {\theta _i}^2\rangle }=\sqrt{1/2}$. According to the central limit theorem^34^, the components of $\textbf{u}(t)$ will have Gaussian PDFs with null average and standard deviation $\sqrt{1/2N}$. The squared polarization $P^2$ is the squared magnitude of such a two-dimensional vector with Gaussian distributed components. This is the same scenario found in the study of optical speckles^28^ where the electric field at a given point results from the sum of electric fields with the same amplitude but randomly distributed phases, the typical scenario of an electric field reflected by a rough surface. The resulting PDF for $P^2$ is found to be exponential, given by

6 $$\begin{aligned} \text {PDF}(P^2) = N e^{-NP^2} ;~~ (N\gg 1). \end{aligned}$$

with $\langle P^2\rangle = SD = 1/N$ in full agreement with the large *N* limits in Eqs.(3-4).

Simple expressions for the PDF of polarization can be derived from the above limiting distributions. For shoals with $N=2$ fish, one finds

7 $$\begin{aligned} \text {PDF}(P) = \frac{2}{\pi \sqrt{1-P^2}} ;~~ (N=2) , \end{aligned}$$

having $\langle P\rangle = 2/\pi \approx 0.636$ and $SD = \sqrt{(\pi ^2-8)/(2\pi ^2)}\approx 0.307$ ($\langle P\rangle / SD = \sqrt{8/(\pi ^2-8)}\approx 2.068$). In the regime of shoals with $N\gg 1$ fish, the polarization distribution assumes the Rayleigh form

8 $$\begin{aligned} \text {PDF}(P) = 2 N P e^{-N P^2} ; ~~(N\gg 1), \end{aligned}$$

having $$\langle P\rangle = \sqrt{\pi /(4N)} \approx 0.886/N^{1/2}$$ and $$SD = \sqrt{(4-\pi )/(4N)}\approx 0.463/N^{1/2}$$ ($$\langle P\rangle / SD = \sqrt{\pi /(4-\pi )}\approx 1.913$$).

Expressions for the exact PDF of *P* and $P^2$ for small $N\ne 2$ can be written in terms of integrals involving Bessel functions^28^.

### Monte Carlo calculations

To obtain numerically the PDFs of fully uncorrelated shoals, we generated $10^6$ configurations for the sum of *N* unit vectors with random orientations. For each configuration, we computed the polarization and its square. Corresponding PDFs were raised using a standard histogram algorithm including all $10^6$ configurations, followed by extracting the relevant statistical quantifiers.

### Supplementary Information

Supplementary Information.

## Data Availability

The data that support the plots within this paper and other findings of this study are available from the corresponding author upon request.
